# Supplementary material for: The interactome of intact mitochondria by cross-linking mass spectrometry provides evidence for coexisting respiratory supercomplexes
Source: Mol Cell Proteomics. 2017 Dec 8;17(2):216–32. doi: 10.1074/mcp.RA117.000470 (PMC5795388; doi:10.1074/mcp.RA117.000470)
Supplement: Supplemental Data [file supp_RA117.000470_133922_0_supp_23978_fzffwf.zip › spectra_annotation/mito_DR_spectra_annotation/96-1-3-1-4-1.pdf]

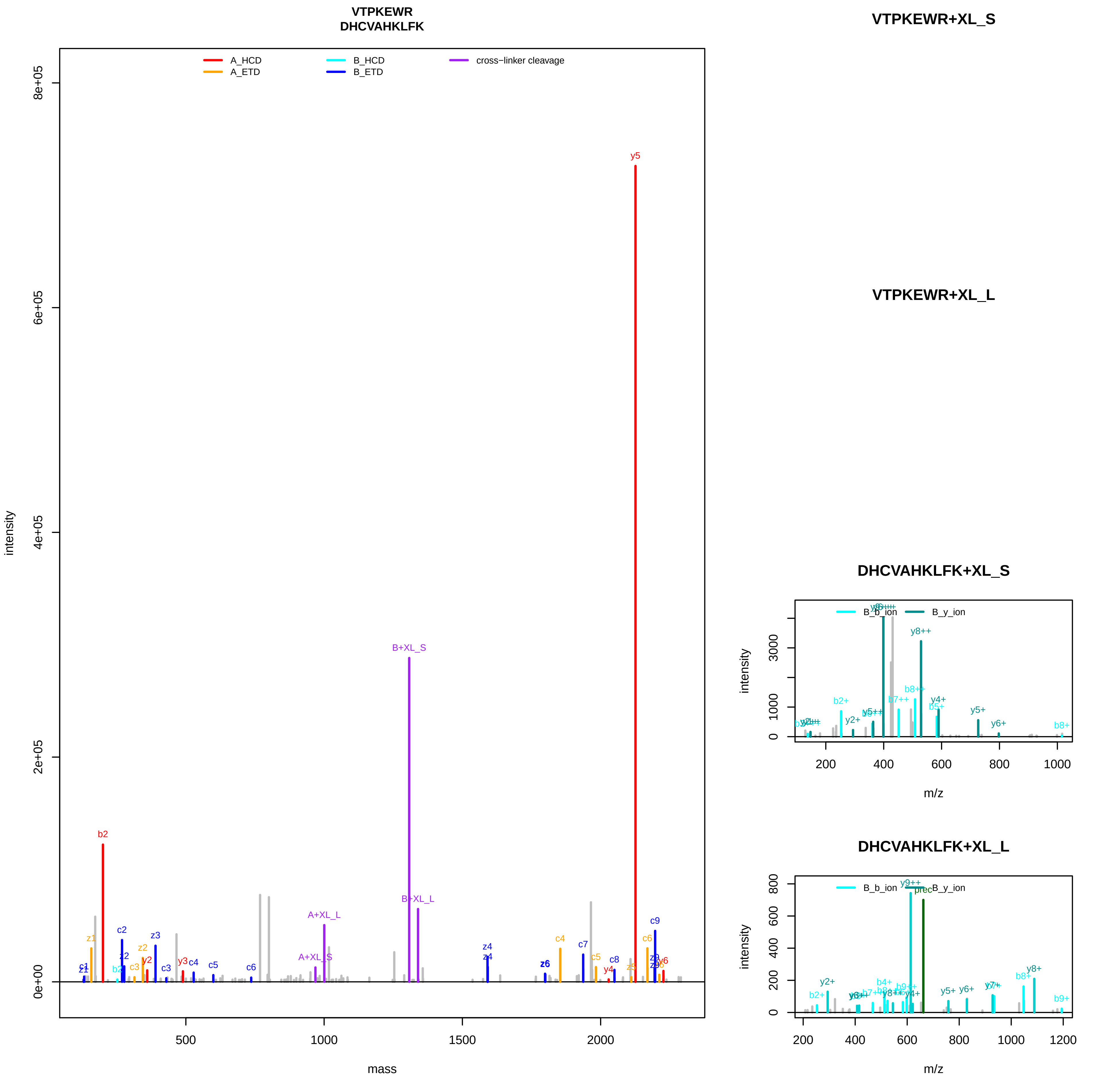

VTPKEWR
DHCVAHKLFK
A_HCD
A_ETD
B_HCD
B_ETD
cross−linker cleavage
intensity
mass
0e+00
2e+05
4e+05
6e+05
8e+05
500
1000
1500
2000
y5
B+XL_S
B+XL_L
A+XL_L
A+XL_S
b2
c2
z3
c9
c4
c7
c6
c5
c8
c3
z1
z2
y2
y3
y4
y6
z4
z6
VTPKEWR+XL_S
VTPKEWR+XL_L
DHCVAHKLFK+XL_S
DHCVAHKLFK+XL_L
B_b_ion
B_y_ion
m/z
y8++
b8++
b7++
y4+
y5+
y6+
b8+
b2+
y2+
y9++
prec
y7+
b9+
y8+
